# Chemical Composition, Antioxidant Activity, and β-Lactamase Inhibitory Potential of Essential Oil from *Micromelum falcatum* (Lour.) Tanaka

**DOI:** 10.3390/molecules31152696

**Published:** 2026-08-03

**Authors:** Yusen Zhang, Zetong Li, Zhuoyi Du, Zi Wang, Xu Liu

**Affiliations:** 1 SDU-ANU Joint Science College, Shandong University, Weihai 264209, China; yusenzhangsdu@outlook.com (Y.Z.); zetongli123@outlook.com (Z.L.); zhuoyi-du@outlook.com (Z.D.); awznbl@outlook.com (Z.W.); 2 Marine College, Shandong University, Weihai 264209, China

**Keywords:** *Micromelum falcatum* (Lour.) Tanaka, essential oil, chemical composition, antioxidant activity, β-lactamase inhibition, molecular docking

## Abstract

*Micromelum falcatum* (Lour.) Tanaka is a medicinal plant widely utilized in traditional Chinese medicine. In this study, the essential oil (EO) was isolated by hydrodistillation using a Clevenger-type apparatus, and its chemical composition and biological activities were systematically investigated. A total of 63 constituents were identified by GC–MS and GC–FID, with bicyclogermacrene (20.52%), γ-palmitolactone (18.16%), humulene (10.55%), caryophyllene (10.09%), and spathulenol (4.50%) representing the major components. Antioxidant capacity was evaluated using DPPH (IC_50_ = 15780 ± 1490 μg/mL), ABTS (IC_50_ = 3440 ± 40 μg/mL), and FRAP assays (Trolox equivalent antioxidant concentration: 734.47 ± 6.82 μmol/g), in which the EO exhibited relatively moderate radical-scavenging and ferric-reducing activities. In addition, the EO demonstrated notable β-lactamase inhibitory activity (IC_50_ = 25.59 ± 4.99 μg/mL). Molecular docking analysis further suggested that compounds such as isospathulenol, globulol, and spathulenol may interact with the enzyme’s active site via hydrogen bonds. These findings indicate that *M. falcatum* EO represents a promising natural source of bioactive compounds with antioxidant and enzyme-inhibitory potential, warranting further pharmacological investigation.

## 1. Introduction

Reactive oxygen species (ROS) are highly reactive molecules generated primarily as byproducts of aerobic metabolism and play essential roles in cellular signaling and physiological homeostasis under normal conditions [1]. However, excessive ROS accumulation, resulting from an imbalance between ROS production and antioxidant defense systems, can disrupt redox homeostasis and induce oxidative stress [2]. Increasing evidence suggests that oxidative stress is closely associated with the pathogenesis of numerous disorders, including Alzheimer’s disease, inflammatory responses, and bacterial infections, through diverse molecular mechanisms [3,4]. Consequently, antioxidant-based interventions have attracted considerable attention as potential strategies for preventing or alleviating oxidative stress-related diseases.

The extensive and prolonged use of synthetic antimicrobial agents has accelerated the emergence of irreversible resistance in numerous bacterial pathogens, including Gram-positive bacteria [5]. Among currently available antibacterial agents, β-lactam antibiotics remain one of the most important therapeutic classes owing to their ability to inhibit bacterial cell wall biosynthesis by targeting penicillin-binding proteins [6]. However, the widespread production of β-lactamases, which enzymatically inactivate β-lactam antibiotics, has substantially compromised their clinical efficacy. Consequently, the co-administration of β-lactam antibiotics with β-lactamase inhibitors has become an established strategy for overcoming bacterial resistance [7]. In this context, natural products exhibiting β-lactamase-inhibitory activity have attracted increasing attention.

Traditionally, synthetic drugs have been regarded as one of the primary therapeutic options for these diseases because of their structurally optimized ligand interactions with specific biological targets [8]. However, increasing concerns about the adverse effects of synthetic agents, together with the continued advancement of natural product research, have revealed that many naturally derived compounds can also interact with multiple biological targets [9]. As complex mixtures of volatile small molecules, essential oils (EOs) are primarily composed of terpenoids, aromatic constituents, and fatty compounds [10]. Owing to their considerable chemical diversity, EOs frequently exhibit broad-spectrum biological activities, including antioxidant, antimicrobial, and enzyme-inhibitory effects, and have potential for industrial-scale production [11,12]. Furthermore, the structural diversity of EO constituents provides an opportunity to investigate potential interactions between selected compounds and β-lactamase using molecular docking, thereby offering preliminary insights into the observed enzyme-inhibitory activity.

**Figure 1:**
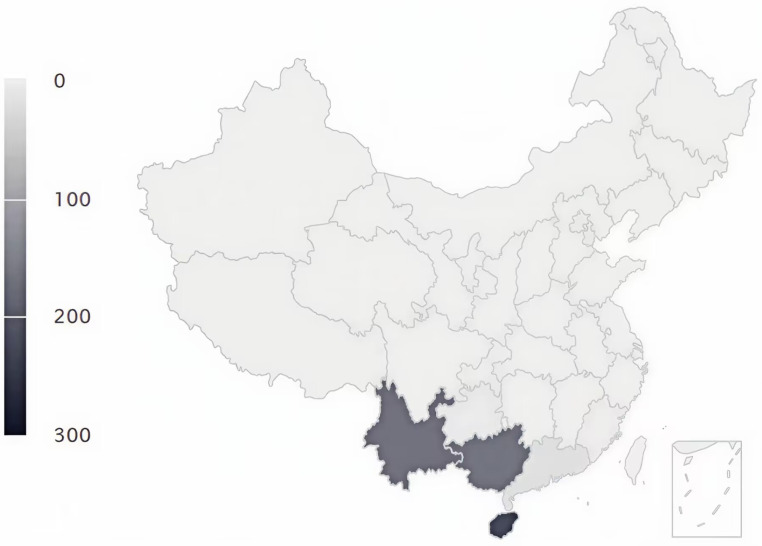
The heatmap of the distribution of *M. falcatum* in China with inset map showing the South China Sea Islands (CVH; https://www.cvh.ac.cn, (assessed on 24th April 2026).

The genus *Micromelum*, belonging to the Rutaceae family, comprises approximately 160 genera and more than 2000 species worldwide [13]. Species in this genus have long been used in traditional medicine and are widely available in the traditional Chinese medicinal market [14]. *Micromelum falcatum* (Lour.) Tanaka, a mangrove-associated medicinal plant distributed in Guangdong, Hainan, Guangxi, and the southeastern Yunnan Province of China (Figure 1), is also found in Vietnam, Laos, Cambodia, and Thailand. Its leaves and stems are traditionally used to promote blood circulation, relieve pain, and treat conditions such as stomachache and rheumatic pain [15,16]. Previous phytochemical investigations have revealed that *M. falcatum* is a rich source of structurally diverse secondary metabolites, particularly prenylated coumarins, lactam alkaloids, and other phenolic constituents with noticeable anti-inflammatory and antibacterial activity. Notably, four new compounds, microminutin B, microminutin C, micromarinate, and secomicromelin, were isolated from the fruits of *M. falcatum,* exhibiting inhibitory activity against the mycelial growth of the fungus-like pathogen *Pythium insidiosum* and demonstrating the promising antimicrobial potential of this species [17,18,19]. Despite these findings, the chemical composition, biological activities, and molecular interaction characteristics of *Micromelum* EOs remain largely unexplored. Therefore, the present study systematically investigated the chemical composition, antioxidant activity, enzyme-inhibitory properties, and molecular docking interactions of the EO derived from *M. falcatum*. This work aims to provide a scientific basis for the further development of *Micromelum* species as potential natural sources of bioactive agents targeting oxidative stress and resistance-associated disorders.

## 2. Results

### 2.1. EO Distillation, GC–FID and GC–MS Analysis

**Figure 2:**
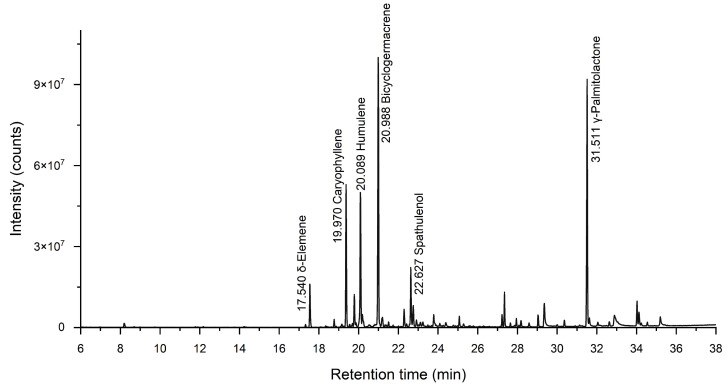
Total ion chromatogram of *M. falcatum* EO.

The *M. falcatum* EO, distilled by hydrodistillation, was a yellow-green, oily substance with a relatively high yield of 0.55 mL/kg (calculated from fresh plant weight), potentially suitable for industrial production. A total of 63 compounds were tentatively identified by GC–MS and GC–FID, accounting for 97.58%. The total ion chromatogram of *M. falcatum* EO reveals the presence of the components, as shown in Figure 2.

As shown in Table 1, the major compounds from *M. falcatum* EO included bicyclogermacrene (20.52%), γ-palmitolactone (18.16%), humulene (10.55%), caryophyllene (10.09%), and spathulenol (4.50%). The chemical distribution of *M. falcatum* EO is demonstrated in Table 1, predominantly characterized by sesquiterpenoids (65.80%) and partly dominated by aliphatic compounds (28.74%) and aromatic compounds (0.43%). Due to the distinctive structure of sesquiterpenoids, these substances with broad-spectrum pharmacological activities can be regarded as a significant source of biologically active compounds in organic and medicinal chemistry.

Bicyclogermacrene, the predominant constituent of *M. falcatum* EO, has previously been reported to exhibit notable biological activities, including cytotoxic effects, which have been further supported by studies involving several tumor-related cell lines [20,21]. γ-Palmitolactone (18.16%), the second most abundant constituent of the EO, has previously been reported as a natural component of EOs and is widely used in the fragrance and flavor industries [22,23]. The presence of these two major constituents may therefore contribute to the biological properties of *M. falcatum* EO. In addition, humulene and caryophyllene, representing 10.55% and 10.09% of the EO, respectively, together with their related derivatives, have been associated with antioxidant effects in previous investigations [24]. These multifunctional bioactivities underscore their relevance for subsequent molecular docking analyses with biologically relevant targets.

Although spathulenol was detected at a comparatively lower level (4.50%), previous studies have demonstrated that this sesquiterpenoid possesses antioxidant, anti-inflammatory, and antibacterial activities. Furthermore, spathulenol exhibited insecticidal activity against *Metopolophium dirhodum*, with a reported LC_50_ value of 1.4 (4.2) mL/L [25], indicating its possible utility as a bioactive pesticidal compound. Notably, the major constituents discussed above are predominantly sesquiterpenes, which collectively accounted for 49.66% of *M. falcatum* EO, highlighting the sesquiterpenoid-rich chemical profile of this species. Besides terpenoid constituents, unsaturated carbonyl compounds were also identified (4.41%). They may further contribute to the observed bioactivities, as compounds containing conjugated carbonyl systems have previously been associated with antioxidant and anti-inflammatory effects [26].

Compared with the report on the EO of the congeneric species *Micromelum integerrimum*, the chemical profile of *M. falcatum* EO was relatively different. The fruit EO of *M. integerrimum* was dominated by monoterpenes, with terpinolene, α-pinene, and β-pinene as the major constituents, whereas the EO of *M. falcatum* was characterized by a sesquiterpene-rich composition, in which bicyclogermacrene, humulene, and caryophyllene were the predominant components. These pronounced differences in chemical composition may be attributed to species-specific metabolic pathways, plant organs (fruit versus aerial parts), geographical origin, developmental stage, and environmental conditions [27]. Such interspecific chemical variation highlights the unique phytochemical characteristics of *M. falcatum* EO and may partly account for the differences in their reported biological activities. Therefore, the chemically diverse composition of *M. falcatum* EO may underlie its broad-spectrum biological properties and supports its further investigation as a source of naturally derived bioactive compounds for industrial and pharmacological applications.

### 2.2. Antioxidant Capacity of M. falcatum EO

The present research evaluated the antioxidant capacity of *M. falcatum* EO using DPPH, ABTS, and FRAP methods from multiple perspectives. As shown in Figure S1 and Table 2, although the *M. falcatum* EO revealed a relatively weak ability to scavenge DPPH free radicals (IC_50_ value: 15,780 ± 1490 μg/mL), its IC_50_ value for ABTS radical scavenging reached 3440 ± 40 μg/mL, higher than *Clerodendrum fortunatum* L. EO (IC_50_ value: 7430 ± 1800 μg/mL) and slightly lower compared to BHT (IC_50_ value: 7.83 ± 1.90 μg/mL) [28]. Compared to the DPPH assay, the ABTS assay confirmed the higher reactivity of the ABTS radical cation, with the calibration curve showing a slope more than five times higher than in the DPPH assay, mainly due to the sterically hindered DPPH radical site, which remains difficult for phenols to access [29]. ABTS test results demonstrate that the inhibition rate increases linearly with EO concentration, exhibiting a dose-dependent relationship. Although involving lower potency relative to synthetic antioxidants, our *M. falcatum* EO demonstrated a relatively more measurable antioxidant capacity in the ABTS assay than in the DPPH assay [30].

In contrast to the DPPH and ABTS assays, which primarily evaluate radical-scavenging behavior via electron-transfer mechanisms, the FRAP assay reflects a sample’s overall ferric ion-reducing capacity. To further assess the antioxidant properties of *M. falcatum* EO, its reducing ability was determined using the FRAP method, yielding a Trolox equivalent antioxidant concentration of 734.47 ± 6.82 μmol/g. This value was markedly higher than those previously reported for *Clerodendrum cyrtophyllum* Turcz. EO and *Clerodendrum fortunatum* L. EO (55.61 ± 2.56 and 23.39 ± 1.58 μmol/g, respectively), indicating a comparatively measurable Fe^3+^-reducing capability [28]. These findings suggest that *M. falcatum* EO may represent a promising natural source of antioxidant-related bioactive constituents.

*M. falcatum* EO possesses a chemically diverse composition in which different classes of constituents may contribute to antioxidant effects through distinct molecular mechanisms. Sesquiterpenes, a group of C_15_ terpenoids biosynthesized from three isoprene units, have previously been associated with the regulation of oxidative stress and inflammatory responses. Representatively, antioxidant sesquiterpenes, such as nerolidol (1.39%) and farnesol isomers (0.44%), have been reported to attenuate neuroinflammation by modulating cellular redox homeostasis [31]. Mechanistically, nerolidol suppresses COX-2-associated activation of the NF-κB signaling pathway, whereas farnesol has been shown to promote Nrf2 activation and nuclear translocation through MEK/ERK1/2-mediated signaling. Subsequent binding of Nrf2 to antioxidant response elements induces the expression of endogenous antioxidant enzymes, including HO-1, SOD, CAT, GPx, and glutathione-related systems, thereby enhancing cellular defense against ROS-mediated oxidative damage [32].

In addition to terpenoid constituents, unsaturated carbonyl compounds identified in *M. falcatum* EO may also contribute to the observed antioxidant properties. Previous studies have demonstrated that compounds containing conjugated carbonyl groups can suppress ROS generation in LPS-stimulated immune cells, suggesting their role in redox regulation. Considering the chemical complexity of essential oils, the observed antioxidant activities are more likely attributable to synergistic or additive interactions among multiple constituents rather than to any single compound. Therefore, the contributions of individual constituents remain speculative and require further investigation through bioassay-guided fractionation and isolated-compound validation.

### 2.3. Enzyme Inhibitory Activity with Molecular Docking Analysis

**Figure 3:**
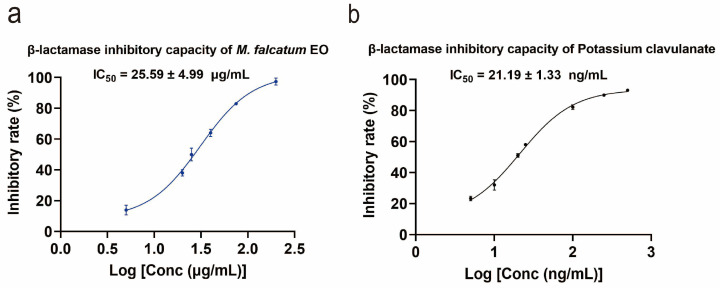
Regression curves of (**a**) *M. falcatum* EO and (**b**) Potassium clavulanate’s anti-β-lactamase capacity.

β-Lactam antibiotics remain among the most widely used classes of antibacterial agents. However, their therapeutic efficacy has been progressively reduced by the widespread emergence of bacterial resistance. Among known resistance mechanisms, β-lactamase-mediated hydrolysis is among the most clinically relevant and well-characterized [33]. Accordingly, the combined use of β-lactam antibiotics with β-lactamase inhibitors has become an effective strategy for restoring antibacterial activity against resistant strains. As illustrated in Figure 3, *M. falcatum* EO exhibited comparatively noticeable β-lactamase inhibitory activity, with an IC_50_ value of 25.59 ± 4.99 μg/mL. This inhibitory effect was stronger than those previously reported for *Clerodendrum cyrtophyllum* Turcz. EO (IC_50_ = 41.34 ± 0.84 μg/mL) and *Clerodendrum fortunatum* L. EO (IC_50_ = 673.50 ± 1.27 μg/mL), indicating that *M. falcatum* EO may represent a promising natural source of β-lactamase-inhibitory constituents.

**Figure 4:**
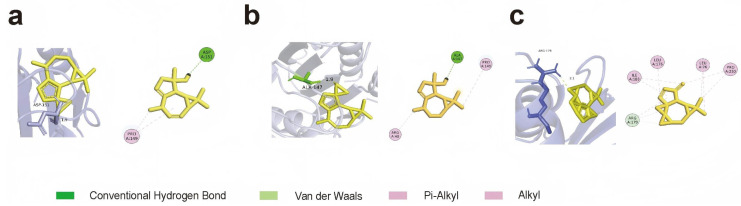
Docking interactions between major compounds and β-lactamase. (**a**) isospathulenol; (**b**) spathulenol; (**c**) globulol. From left to right, the partial 3D and 2D images are presented. In the 3D images, blue cartoons represent β-lactamase protein, yellow sticks represent compounds from *M. falcatum* EO, and purple sticks represent amino acid residues bonded with small ligands. The names of the amino acid residues and the lengths of the hydrogen bonds are presented in each figure. In the 2D images, green dots represent hydrogen bonding, light green dots represent Van der Waals, red dots represent unknown force, and pink and purple dots represent hydrophobic interactions.

These results indicate that the tentatively identified major components of *M. falcatum* EO may exhibit relative inhibitory potency against β-lactamases; we therefore conducted a molecular docking study to probe their binding affinity. The present experiment conducted molecular docking analysis on the compounds in *M. falcatum* EO with contents greater than 1%, and the results are shown in Table 3. As shown in the figures, chemical components such as bicyclogermacrene (20.52%), γ-palmitolactone (18.16%), humulene (10.55%), and caryophyllene (10.09%) exhibited noticeable binding energies of −5.95, −5.34, −6.10, and −5.47 kcal/mol, respectively. Notably, several compounds, including spathulenol (Residue: ALA147, −6.04 kcal/mol; Bond lengths: 1.9 Å; RMSD: 0.22 Å), isospathulenol (Residue: ASP−151, −5.20 kcal/mol; Bond lengths: 1.9 Å; RMSD: 0.47 Å), and globulol (Residue: ARG-179, −6.10 kcal/mol; Bond lengths: 2.1 Å; RMSD: 2.14 Å), demonstrated hydrogen bonding interactions with the active site of β-lactamase, shown in Figure 4. Additionally, previous studies have reported that spathulenol, isospathulenol, and globulol, constituents commonly detected in plant EOs, may exhibit positive antibacterial activity [34,35,36]. Therefore, the docking analysis suggests that several tentatively identified constituents may interact with amino acid residues within or near the β-lactamase active site, warranting in vivo experimental verification. However, further studies are required to elucidate the respective contributions of individual constituents and the potential synergistic or additive effects among multiple components.

## 3. Materials and Methods

### 3.1. Plant Material and Essential Oil Extraction

Fresh branches and leaves of *Micromelum falcatum* (Lour.) Tanaka were collected from Qinzhou City, Guangxi Province, China (108°40′7.57″ E, 22°2′28.66″ N) on 5 September 2024. The plant material was taxonomically authenticated by Prof. Hong Zhao, and a voucher specimen (EO2404) was deposited at the Center for Bioscience Analysis and Testing, Shandong University, Weihai, China. Collection of the wild plant material required no specific permission, and the investigated species is not included in either the List of Nationally Protected Wild Plants (2021) or the CITES Appendices (2023).

Fresh leaves and stems (1.00 kg) were mechanically homogenized into small fragments and transferred into a 5 L round-bottom flask containing 2.5 L of ultrapure water. Essential oil (EO) was subsequently isolated by hydrodistillation using a Clevenger-type apparatus (Synthware, Chongqing, China) for 5 h with diethyl ether as the extraction solvent. The EO was stored in sealed glass vials under air at 4 °C in dark conditions.

### 3.2. GC–MS and GC–FID Analysis

The *M. falcatum* EO components and their contents were analyzed by gas chromatography–mass spectrometry (GC–MS) and gas chromatography–flame ionization detector (GC–FID), with modifications as described in previous papers [28]. GC–MS analysis was carried out on an Agilent 7890 gas chromatograph coupled to an Agilent 5975C mass spectrometer (Agilent Technologies, Santa Clara, CA, USA) and fitted with an HP-5MS fused-silica capillary column (30 m × 0.25 mm i.d., 0.25 μm film thickness). Helium was employed as the carrier gas at a constant flow rate of 1.1 mL/min. The oven temperature was programmed as follows: initial temperature 50 °C (held for 4 min), increased to 280 °C at 6 °C/min, and finally held at 280 °C for 3 min. The injector temperature and interface temperature were set to 260 °C and 280 °C, respectively. Mass spectrometry conditions were as follows: quadrupole temperature of 150 °C, mass scan range of 25-500 amu, scan rate of 4.0 scans/s, and electron ionization (EI) mode at 70 eV. 1% (*w*/*v*) sample solution in *n*-hexane was prepared, and 0.3 μL was injected in splitless mode. GC–FID analysis was used on an Agilent 7890 gas chromatograph equipped with an HP-5 fused-silica capillary column (30 m × 0.25 mm, 0.25 μm film thickness; Agilent Technologies, Santa Clara, CA, USA). Helium was used as the carrier gas at a flow rate of 1.1 mL/min. The oven temperature program was identical to that used in the GC–MS analysis. The injector and detector temperatures were maintained at 260 °C and 305 °C, respectively. Compound identification was based on comparison of mass spectra with the NIST/EPA/NIH 2023 Mass Spectral Library and by comparison of calculated Kováts retention indices (RIs) with reference values. The RIs were determined using a homologous series of *n*-alkanes (C_8_–C_30_) analyzed under the same chromatographic conditions, with linear interpolation on the HP-5MS column. Data processing was performed using Agilent MassHunter Qualitative Analysis 10.0.

### 3.3. Antioxidant Capacity Analysis

#### 3.3.1. FRAP Test

The ferric-reducing antioxidant power (FRAP) method was carried out with minor modifications based on a previously reported procedure [37]. The FRAP method working solution was freshly prepared by mixing 0.3 M acetate buffer (pH 3.6), 10 mM TPTZ solution, and 20 mM FeCl_3_ solution at a volume ratio of 10:1:1, followed by dilution with ethanol (approximately 40–50 fold).

A total of 200 μL of the FRAP reagent was transferred into each well of a 96-well microplate, and 50 μL of EO solution at concentrations of 100, 250, 500, 1000, 2000, and 5000 μg/mL was added. Trolox solutions (0.25 mg/mL) at different volumes (0, 2.5, 5, 10, 15, and 20 μL) were used to generate the calibration curve, while ethanol was used as the blank control instead of the EO solution. Absorbance was recorded at 593 nm using a microplate reader (Epoch, Biotech, Minneapolis, MN, USA) after incubation at 37 °C for 30 min.

The antioxidant capacity of each sample was expressed as Trolox equivalent antioxidant capacity (TEAC). Based on the Trolox standard curve, the amount of equivalent Trolox (X, nmol) corresponding to the absorbance of each EO sample was obtained. The FRAP value was then calculated according to the equation 20X/Y (μmol/g), where Y represents the EO concentration (μg/mL). All measurements were independently performed three times, and the results are presented as mean values.

#### 3.3.2. ABTS Test

The ABTS radical scavenging activity was evaluated according to previously published methods with slight modifications [28,37]. Briefly, EO samples were prepared in ethanol at concentrations of 0.1, 0.5, 1, 2.5, 5, and 10 mg/mL. ABTS stock solution (7.4 mM) was mixed with an equal volume of 2.6 mM potassium persulfate solution and kept in the dark at room temperature for at least 12 h to generate the ABTS^•+^ radical. In each well of a 96-well plate, 200 μL of diluted ABTS^•+^ solution was mixed with 50 μL of the EO sample. Trolox was used as the positive control, whereas ethanol served as the blank.

After 7 min of reaction, absorbance was measured at 734 nm using a microplate reader (Epoch, Biotech, Minneapolis, MN, USA). The scavenging capacity was calculated using the equation below:

$$RSC\%\;=(\frac{A_{0}-A}{A_{0}})\times \;100\%$$

*RSC*% represents the “radical scavenging activity” of the ABTS^•+^ radical, *A*_0_ and *A* are the absorbance of 200 μL diluted ABTS^•+^ solution mixed with 50 μL ethanol sample solution at 734 nm, respectively. IC_50_ was then calculated using GraphPad Prism 9.5.

#### 3.3.3. DPPH Test

DPPH radical scavenging activity was determined following a previously described protocol with minor modifications [18]. A 0.17 mM DPPH solution was freshly prepared in ethanol prior to the experiment. EO samples were diluted with methanol to final concentrations of 1, 2, 5, 10, 20, 40, and 80 mg/mL.

For each reaction, 200 μL of DPPH solution was mixed with 50 μL of the EO sample in a 96-well microplate. Trolox or BHT was employed as the positive control. The control group contained DPPH solution and ethanol only, whereas the sample blank consisted of EO solution mixed with ethanol without DPPH. After incubation in the dark for 30 min at room temperature, absorbance was determined at 516 nm using a microplate reader (Epoch, Biotech, Minneapolis, MN, USA), and then calculated IC_50_ values were determined using the equation:

$$RSC\%\;=(1-\frac{A_{sample}-A_{blank}}{A_{control}})\times \;100\%$$

*A_Sample_* and *A_Control_* represent the absorbance of a 250 μL system including EO with a series of concentrations and DPPH control solution, with *A_blank_* representing an ethanol blank system without DPPH. IC_50_ was calculated using GraphPad Prism 9.5.

### 3.4. Anti-β-Lactamase Test

β-Lactamase inhibitory activity was evaluated according to a previously described protocol with minor modifications [28]. The EO samples were prepared in ethanol at final concentrations of 5, 20, 25, 40, 75, and 200 μg/mL. Potassium clavulanate served as the reference inhibitor. In total, 20 μL of sample solution was mixed with 20 μL of β-lactamase solution (1000 U/mL) and 110 μL of phosphate-buffered saline (PBS, 50 mM, pH 7.0) in a 96-well microplate, then pre-incubated at 30 °C for 10 min. Subsequently, 50 μL of Nitrocefin solution (0.1 mg/mL) was introduced to initiate the enzymatic reaction, and the mixture was further incubated at 30 °C for 10 min. Absorbance was then recorded at 489 nm using a microplate reader. All measurements were independently performed in triplicate. The β-lactamase inhibition rate was calculated as follows:

$$Inhibition\%\;=\;(1-\frac{A_{s}-A_{e}}{A_{sb}-A_{b}})\;\times \;100\%$$

*A_s_* and *A_e_* are the absorbance of the sample containing EO and enzymatic determination, with *A_sb_* and *A_b_* representing the blank reaction absorbance of the sample and the blank reaction absorbance, respectively. The IC_50_ value was calculated through nonlinear regression by GraphPad Prism 9.5.

### 3.5. Molecular Modeling Analysis

#### 3.5.1. Molecular Docking Simulation

Molecular modeling was performed to investigate the interactions between the essential oil components and the target enzymes. The crystal structure of β-lactamase from *Escherichia coli* (PDB ID: 7QOR) was retrieved from the RCSB Protein Data Bank (http://www.rcsb.org/). Additionally, we selected 2-{1-[4-(12-amino-3-chloro-6,7,10,11-tetrahydro-7,11-dimethylcycloocta[b]quinolin-9-yl)butyl]-1H-1,2,3-triazol-4-yl}-N-(4-hydroxy-3-methoxyphenethyl)acetamide and 2-amino-2-hydroxymethyl-propame-1,3-diol (PubChem CID: 156612856 and 3777159) as the co-crystallized ligand used for validation in AChE and β-lactamase, respectively. The obtained RMSD values were 0.05 and 0.47 Å, which were below the generally accepted threshold of 2.0 Å, indicating that the docking protocol can reliably reproduce the experimentally observed binding mode and is therefore suitable for subsequent molecular docking analyses.

#### 3.5.2. Protein and Ligand Preparation

Prior to docking, all receptor structures were prepared using AutoDock Tools (version 1.5.7). Specifically, co-crystallized ligands and coordinated water molecules were removed from the binding pockets to prevent steric interference. Polar hydrogen atoms were then added to the protein structures to ensure appropriate protonation states of ionizable residues. The three-dimensional structures of the major essential oil components (acting as ligands) were retrieved from the ChemSpider database (https://www.chemspider.com/), a reliable public repository for chemical compounds.

#### 3.5.3. Docking Protocol

Semi-flexible molecular docking was performed using AutoDock Vina. For each target protein, the grid box was centered on the active site, at x = −8.905, y = −2.095, and z = 89.421, with dimensions of 120 (x) × 126 (y) × 118 (z) grid points and a spacing of 0.889 Å to encompass the entire binding pocket. To enable a comprehensive conformational search, the exhaustiveness parameter was increased to 25. A maximum of 10 binding poses were generated per ligand, allowing for a robust sampling of potential binding modes.

#### 3.5.4. Visualization and Analysis

The resulting complexes were evaluated based on their calculated binding affinities (expressed in kcal/mol) and the specific intermolecular interactions formed between each ligand and the receptor. Notably, hydrogen bond interactions were identified and further analyzed. All binding modes and hydrogen bond networks were visualized and interpreted using PyMOL (version 3.1).

### 3.6. Statistical Analysis

All our antioxidant assays (DPPH, ABTS, and FRAP) and β-lactamase inhibition experiments were conducted in triplicate, and data are presented as mean ± standard deviation (SD). IC_50_ values (anti-β-lactamase activity; DPPH and ABTS scavenging activities) were obtained by nonlinear regression analysis using GraphPad Prism 9.5.

## 4. Conclusions

*Micromelum falcatum* (Lour.) Tanaka is regarded as a renowned plant with a long-standing record of use in traditional Chinese medicine and a wide market presence. The present study provides the first report on the chemical composition and bioactivities of *Micromelum falcatum* (Lour.) Tanaka essential oil (EO) from China. Based on the overall results using GC–MS and GC–FID, *M. falcatum* EO exhibited a relatively high yield (0.55 mL/kg) and a chemically diverse composition comprising 63 tentatively identified constituents including bicyclogermacrene, γ-palmitolactone, humulene, caryophyllene, and spathulenol. The positive outcomes obtained from the DPPH, ABTS, and FRAP assays indicate that *M. falcatum* EO possesses measurable antioxidant properties, providing experimental support for its further exploration as a naturally derived antioxidant source. In addition, the in vitro enzyme inhibition results, together with molecular docking analysis, suggest that several tentatively identified constituents, including sesquiterpenoids and related compounds, may be associated with the observed β-lactamase inhibitory activity. Since β-lactamase-mediated hydrolysis is one of the major mechanisms underlying bacterial resistance to β-lactam antibiotics, the inhibitory activity observed in the present study suggests that *M. falcatum* EO may represent a potential source of naturally occurring β-lactamase inhibitors. Nevertheless, whether this inhibitory activity can restore antibiotic susceptibility in resistant bacterial strains requires further toxicity evaluation, antimicrobial susceptibility testing, and microbiological validation using combination susceptibility assays.

These findings collectively indicate that *M. falcatum* EO contains chemically diverse constituents associated with antioxidant and enzyme-inhibitory effects. Considering its relatively abundant EO yield and observable bioactivities, future studies should compare the chemical composition and biological activities of essential oils isolated from different organs of *M. falcatum*, including leaves, stems, fruits, and flowers, to better understand organ-specific metabolic variation. Additionally, investigations involving compound isolation, mechanistic validation, toxicity evaluation, and in vivo models are warranted to clarify its pharmacological relevance and possible industrial applications.

## Figures and Tables

**Table 1:** Chemical composition of EO distilled from *M. falcatum.*

No.	RT	Compound (RRI, MS)	RI_Calc_	RI_Ref_	R. Match	Area (%)	CAS ID	Type
1	8.190	β-Pinene	975	975–983	944	0.42	127-91-3	MT
2	14.219	Methyl salicylate	1197	1187–1197	868	0.13	119-36-8	Arom
3	17.540	δ-Elemene	1339	1336–1340	940	3.35	20307-84-0	SQT
4	18.344	Isoledene	1376	1373–1377	830	0.10	95910-36-4	SQT
5	18.767	β-Elemene	1395	1391–1395	892	0.56	515-13-9	SQT
6	19.158	α-Gurjunene	1413	1407–1411	848	0.32	489-40-7	SQT
7	19.370	Caryophyllene	1423	1415–1423	957	10.09	87-44-5	SQT
8	19.549	Calarene	1433	1429–1435	770	0.20	17334-55-3	SQT
9	19.687	α-Guaiene	1440	1439–1441	879	0.34	3691-12-1	SQT
10	19.782	Aromandendrene	1444	1442–1446	941	2.35	489-39-4	SQT
11	19.867	Selina-5,11-diene	1449	1447	737	0.31	52026-55-8	SQT
12	20.089	Humulene	1459	1451–1457	942	10.55	6753-98-6	SQT
13	20.184	Alloaromadendrene	1462	1459–1463	764	1.48	25246-27-9	SQT
14	20.522	γ-Muurolene	1479	1474–1480	815	0.23	30021-74-0	SQT
15	20.575	γ-Selinene	1481	1473–1485	692	0.11	515-17-3	SQT
16	20.766	(*E*)-β-Ionone	1490	1489–1493	693	0.09	79-77-6	Aliph
17	20.819	β-Guaiene	1493	1489–1497	776	0.19	88-84-6	SQT
18	20.988	Bicyclogermacrene	1501	1491–1507	841	20.52	24703-35-3	SQT
19	21.199	α-Farnesene	1512	1506–1510	796	1.16	502-61-4	SQT
20	21.337	γ-Cadinene	1519	1511–1515	851	0.11	39029-41-9	SQT
21	21.400	Nootkatene	1522	1516–1522	720	0.10	5090-61-9	SQT
22	21.506	δ-Cadinene	1527	1524	852	0.31	483-76-1	SQT
23	21.739	β-Vetivenene	1540	1527–1553	732	0.09	27840-40-0	SQT
24	22.289	(*E*)-Nerolidol	1567	1561–1567	931	1.39	40716-66-3	SQT-O
25	22.416	Maaliol	1574	1570–1578	725	0.27	527-90-2	SQT-O
26	22.627	Spathulenol	1584	1578–1584	751	4.50	6750-60-3	SQT-O
27	22.754	Globulol	1591	1589–1593	800	1.92	489-41-8	SQT-O
28	22.913	Guaiol	1599	1593–1599	829	0.65	489-86-1	SQT-O
29	23.018	Humulene epoxide I	1605	1601–1607	724	0.19	19888-33-6	SQT-O
30	23.114	Rosifoliol	1610	1595–1605	832	0.45	63891-61-2	SQT-O
31	23.241	Humulene epoxide II	1616	1604–1608	818	0.64	19888-34-7	SQT-O
32	23.579	Ledene oxide-(II)	1635	1631	711	0.09	882187-44-2	SQT-O
33	23.685	Caryophylladienol II	1640	1633–1641	776	0.11	38284-26-3	SQT-O
34	23.780	Isospathulenol	1645	1643–1647	794	1.23	88395-46-4	SQT-O
35	23.907	α-Vetivol	1652	1656	697	0.18	57422-86-3	SQT-O
36	24.087	Neointermedeol	1662	1658–1662	765	0.41	5945-72-2	SQT-O
37	24.267	Ylangenol	1672	1666	667	0.15	41610-69-9	SQT-O
38	24.393	Aromadendrene epoxide	1678	1672–1678	786	0.55	94020-95-8	SQT-O
39	24.774	Acorenone B	1699	1700–1702	753	0.16	21653-33-8	SQT-O
40	25.070	*n*-Pentadecanal	1716	1712–1718	952	0.78	2765-11-9	Aliph
41	25.282	trans-Farnesol	1728	1722	775	0.28	106-28-5	SQT-O
42	25.567	(2*E*, 6*Z*)-Farnesol	1744	1742	754	0.16	3879-60-5	SQT-O
43	26.287	Anthracene	1784	1786	762	0.12	120-12-7	Arom
44	27.228	Neophytadiene	1840	1832–1842	939	0.82	504-96-1	Aliph
45	27.344	Hexahydrofarnesyl acetone	1847	1840–1848	936	2.39	502-69-2	Aliph
46	27.640	(*Z*)-9-Hexadecen-1-ol	1864	1863–1865	730	0.28	10378-01-5	Aliph
47	27.862	Benzyl salicylate	1877	1862–1876	799	0.18	118-58-1	Arom
48	27.947	(*E*)-11-Hexadecen-1-ol	1882	1877	745	0.56	61301-56-2	Aliph
49	28.074	(8*Z*, 11*Z*)-Heptadecadienal	1890	1886	826	0.11	56797-42-3	Aliph
50	28.180	Methyl (7*E*, 10*E*)-7,10-hexadecadienoate	1896	1891–1897	794	0.47	17364-31-7	Aliph
51	28.582	Farnesyl acetone	1921	1914–1924	801	0.37	1117-52-8	Aliph
52	29.036	Isophytol	1949	1946–1950	917	0.83	505-32-8	DT
53	29.999	13-Octadecenal	2010	2014	679	0.17	56554-90-6	Aliph
54	30.358	Geranyllinalool	2033	2034	826	0.46	1113-21-9	DT
55	31.131	(*Z*)-9-Octadecenenitrile	2083	2084	796	0.13	112-91-4	Aliph
56	31.511	γ-Palmitolactone	2109	2104–2106	960	18.16	730-46-1	Aliph
57	31.628	Phytol	2117	2109–2119	873	0.90	150-86-7	DT
58	32.051	Oleic acid	2145	2130–2152	728	0.44	112-80-1	Aliph
59	32.622	1-Docosene	2185	2194	700	0.33	1599-67-3	Aliph
60	34.029	Methyl 11,14,17-eicosatrienoate	2283	2275–2283	713	1.90	97016-84-7	Aliph
61	34.124	(*Z*)-9-Octadecen-4-olide	2290	2287	738	1.21	59303-50-3	Aliph
62	34.500	Methyl 11,14-Eicosadienoate	2299	2294–2304	727	0.25	2463-02-7	Aliph
63	34.547	γ-Stearolactone	2321	2319	746	0.28	502-26-1	Aliph
		Monoterpenes (MT)				0.42		
		Sesquiterpenes (SQT)				52.47		
		Sesquiterpenoids (SQT-O)				13.33		
		Diterpene (DT)				2.19		
		Aliphatic compounds (Aliph)				28.74		
		Aromatic compounds (Arom)				0.43		
		Total identified				97.58		

Concentration calculated from total ion chromatogram; RT: Retension time. RI_Calc_: Arithmetic index calculated on an HP-5MS column. RI_Ref_: Acceptable RI deviation range obtained from mass spectral database. R. Match: Reverse Match (0–1000), GC–MS match factor obtained from mass spectral library searching, reflecting the similarity between the experimental mass spectrum and the reference spectrum in the database. CAS ID: Chemical Abstracts Service Registry Number for compounds. Identification method based on the relative retention indices (RRI) of authentic compounds on the HP-5MS column, and identified based on computer matching of the mass spectra (MS) with NIST/EPA/NIH 2023 Mass Spectral Database and comparison with literature data.

**Table 2:** Antioxidant activities were measured by DPPH and ABTS assays, and by the FRAP assay.

Tested Samples	DPPH (IC_50_)	ABTS (IC_50_)	FRAP (µmol/g)
*M. falcatum* EO	15,780 ± 1490 μg/mL	3440 ± 40 μg/mL	734.47 ± 6.82
BHT	45.03 ± 1.56 μg/mL	4.51 ± 0.21 μg/mL	-

**Table 3:** The docking results of *β*-lactamase with major compounds (content > 1%) of *M. falcatum* EO.

Compound	Binding Energy (kcal/mol)	Van der Waals Hydrogen Bond Desolvation Energy (kcal/mol)	Ligand Efficiency (kcal/mol)
δ-Elemene	−5.64	−5.94	−0.38
Caryophyllene	−5.47	−5.47	−0.36
Aromandendrene	−6.26	−6.25	−0.42
Humulene	−6.10	−6.09	−0.41
Alloaromadendrene	−5.30	−5.29	−0.35
Bicyclogermacrene	−5.95	−5.95	−0.40
α-Farnesene	−4.79	−6.58	−0.32
(*E*)-Nerolidol	−5.86	−7.34	−0.37
Spathulenol	−6.04	−6.33	−0.38
Globulol	−6.10	−6.12	−0.38
Hexahydrofarnesyl acetone	−4.50	−6.56	−0.24
γ-Palmitolactone	−5.34	−7.15	−0.30
Methyl 11,14,17-eicosatrienoate	−2.48	−6.06	−0.11
(*Z*)-9-Octadecen-4-olide	−6.83	−7.12	−0.38
Isospathulenol	−5.20	−5.43	−0.33

## Data Availability

The data presented in this study are available from the corresponding author upon reasonable request.

## Supplementary Materials

The following supporting information can be downloaded at: https://www.mdpi.com/article/10.3390/molecules31152696/s1, Figure S1: Regression curves of (a) *M. falcatum* EO and (b) BHT’s DPPH scavenging activity and (c) *M. falcatum* EO and (d) BHT’s ABTS scavenging activity; Blank Report; Compounds Identification Report.

## Abbreviations

The following abbreviations are used in this manuscript:

ABTS	2,2′-azino-bis(3-ethylbenzothiazoline-6-sulfonic acid)
BHT	Butylated hydroxytoluene
DPPH	2,2-diphenyl-1-picrylhydrazyl
DTNB	5,5′-dithiobis(2-nitrobenzoic acid)
EO	Essential oil
EOs	Essential oils
FRAP	Ferric-reducing antioxidant power
GC–FID	Gas chromatography–flame ionization detector
GC–MS	Gas chromatography–mass spectrometry
IC_50_	Half-maximal inhibitory concentration
MI	Myocardial infarction
OMPs	Outer membrane proteins
PBS	Phosphate-buffered saline
RIs	Retention indices
ROS	Reactive oxygen species
RSC%	Radical scavenging capacity
*M. falcatum*	*Micromelum falcatum* (Lour.) Tanaka
TIC	Total ion chromatogram
