# Supplementary material for: Chemical Composition, Antioxidant Activity, and β-Lactamase Inhibitory Potential of Essential Oil from Micromelum falcatum (Lour.) Tanaka
Source: Molecules. 2026 Aug 3;31(15):2696. doi: 10.3390/molecules31152696 (PMC13467851; doi:10.3390/molecules31152696)
Supplement: Supplementary file 1 [file molecules-31-02696-s001.zip › blank Report.pdf]

Analysis Report

Sample Information

SampleName	KB	DataFilePath	F:\deanwhite\6-科研资料\研究工作\挥发油\4-数据处理\20250110\【Test data】\KB.D
SampleID		AcqTime_Local	2025/1/13 14:30:56 (UTC+08:00)
InstrumentName	GCMS-5975C	MethodPath_Acquisition	2025做样测试.M
MSType	Q	Version_AcqSW	
InjectionVolume	0	IRMStatus	
SamplePosition	3	MethodPath_Analysis	F:\deanwhite\6-科研资料\研究工作\挥发油\4-数据处理\20250110\【Test data】\KB.D\Results\Qual\Version4\deanwhite 240123.m
PlatePosition		TargetSourcePath	D:\MassHunter\Library\NIST20.L;D:\MassHunter\PCDL\Pesticide_Example.cdb;D:\MassHunter\PCDL\Test_AM_PCDL.cdb
Operator	MassHunter GC/MS Translator	ResultSummary	

Sample Chromatograms

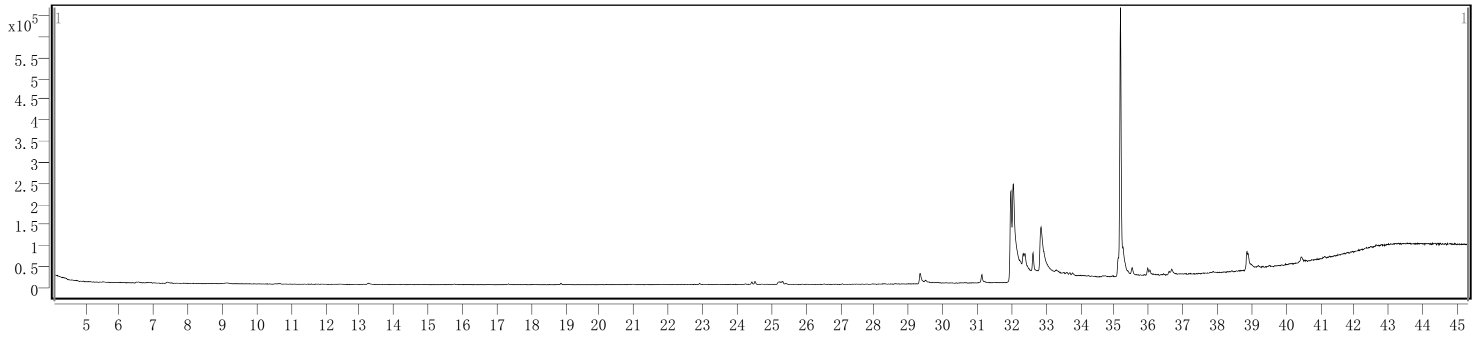

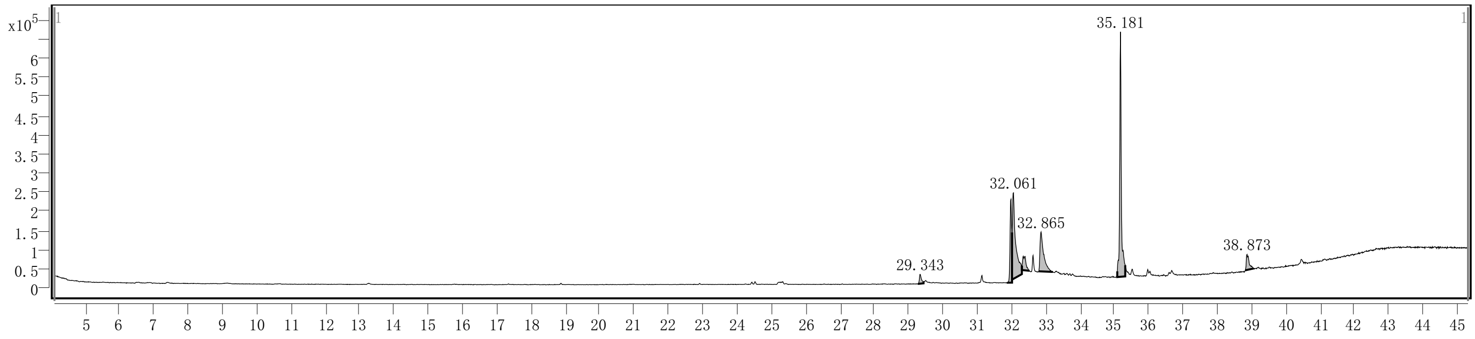

Chromatogram Peaks

Peak	StartRT	RT	EndRT	Height	Area	AreaPct	SNR
1	29.273	29.343	29.438	24382	103075	4.74	
2	31.892	31.987	32.019	217810	710183	32.68	
3	32.019	32.061	32.305	225007	1507278	69.36	
4	32.305	32.347	32.523	36577	252249	11.61	
5	32.797	32.865	33.208	102985	767826	35.33	
6	35.086	35.181	35.319	640704	2173056	100.00	
7	38.821	38.873	39.063	40600	234768	10.80	

Compound Summary

Cpd	CpdName	Formula	RT	Mass	CAS	IDSourceBest	Score_ID	Score_Lib	Score_DB	Score_MFG	Algorithm
1	n-Hexadecanoic acid	C16 H32 O2	29.343		57-10-3	Lib searching	89.83	89.83			integral
2	Linoelaidic acid	C18 H32 O2	31.987		506-21-8	Lib searching	95.02	95.02			integral
3	9-Octadecenoic acid, (E)-	C18 H34 O2	32.061		112-79-8	Lib searching	73.64	73.64			integral
4	Octadecanoic acid	C18 H36 O2	32.347		57-11-4	Lib searching	67.51	67.51			integral
5	Phenol, 4,4'-(1-methylethylidene)bis-	C15 H16 O2	32.865		80-05-7	Lib searching	95.45	95.45			integral
6	9-Octadecenamide, (Z)-	C18 H35 N O	35.181		301-02-0	Lib searching	93.94	93.94			integral
7	?	C6 H12 O	38.873			Lib searching	100.00				integral

MassHunter Qual 10.0
(End of Report)
