# Supplementary material for: Chemical Composition, Antioxidant Activity, and β-Lactamase Inhibitory Potential of Essential Oil from Micromelum falcatum (Lour.) Tanaka
Source: Molecules. 2026 Aug 3;31(15):2696. doi: 10.3390/molecules31152696 (PMC13467851; doi:10.3390/molecules31152696)
Supplement: Supplementary file 1 [file molecules-31-02696-s001.zip › Compounds Identification Report.pdf]

Analysis Report

Agilent | Trusted Answers

Sample Information

SampleName	E02404	DataFilePath	D:\GCMS\E02404.D
SampleID		AcqTime_Local	2025/1/14 11:54:58 (UTC+08:00)
InstrumentName	GCMS-5975C	MethodPath_Acquisition	2025TEST.M
MSType	Q	Version_AcqSW	
InjectionVolume	0	IRMStatus	
SamplePosition	7	MethodPath_Analysis	D:\GCMS\E02404.D\Results\Qual\Version4\zys.m
PlatePosition		TargetSourcePath	D:\MassHunter\Library\NIST20.L;D:\MassHunter\PCDL\Pesticide_Example.cdb;D:\MassHunter\PCDL\Test_AM_PCDL.cdb
Operator	MassHunter GC/MS Translator	ResultSummary	

Sample Chromatograms

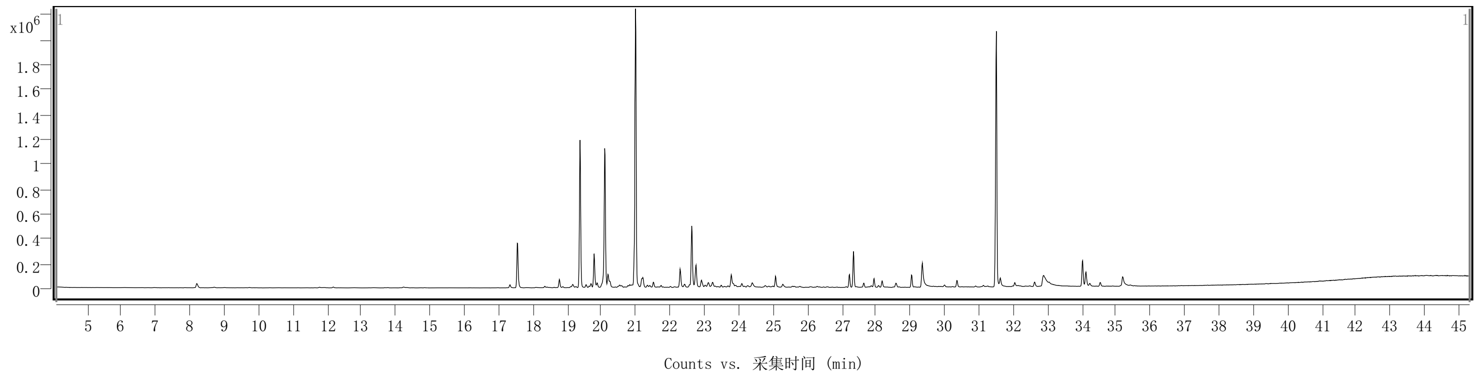

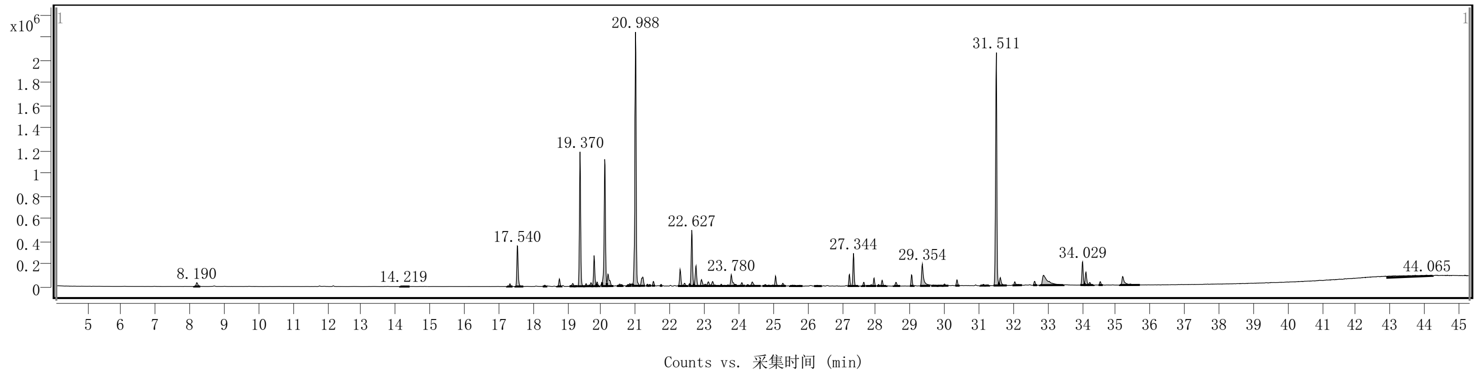

Chromatogram Peaks

Peak	StartRT	RT	EndRT	Height	Area	AreaPct	SNR
1	8.116	8.190	8.294	33644	123501	2.05	
2	14.124	14.219	14.399	6017	39496	0.66	
3	17.244	17.328	17.401	24112	66633	1.11	
4	17.487	17.540	17.715	357645	982004	16.30	
5	18.284	18.344	18.386	11610	29704	0.49	
6	18.703	18.767	18.830	65284	164902	2.74	
7	19.063	19.158	19.211	25025	95064	1.58	
8	19.211	19.253	19.294	7608	20753	0.34	
9	19.299	19.370	19.486	1182016	2962690	49.18	
10	19.486	19.549	19.602	22194	59431	0.99	
11	19.602	19.687	19.729	30314	100847	1.67	
12	19.729	19.782	19.846	270031	690460	11.46	
13	19.846	19.867	19.941	36241	90348	1.50	
14	20.015	20.089	20.152	1114740	3095872	51.39	
15	20.152	20.184	20.343	104172	433341	7.19	
16	20.444	20.522	20.565	16986	67018	1.11	
17	20.565	20.575	20.634	12134	31065	0.52	
18	20.723	20.766	20.787	12839	27290	0.45	
19	20.787	20.819	20.850	20046	55545	0.92	
20	20.914	20.988	21.104	2232611	6023910	100.00	
21	21.104	21.199	21.264	76764	341034	5.66	
22	21.301	21.337	21.369	14589	33698	0.56	
23	21.369	21.400	21.450	11662	29041	0.48	
24	21.469	21.506	21.557	37807	91963	1.53	
25	21.697	21.739	21.769	12858	25932	0.43	
26	22.226	22.289	22.373	145320	407341	6.76	
27	22.373	22.416	22.511	23987	77830	1.29	
28	22.574	22.627	22.691	491592	1321923	21.94	
29	22.691	22.754	22.849	176787	564629	9.37	
30	22.849	22.913	22.976	56343	190247	3.16	
31	22.976	23.018	23.061	15935	54940	0.91	
32	23.061	23.114	23.167	37087	133398	2.21	
33	23.167	23.241	23.442	39026	189049	3.14	
34	23.442	23.484	23.526	15911	38236	0.63	
35	23.526	23.579	23.643	6550	25421	0.42	
36	23.643	23.685	23.727	10625	31419	0.52	
37	23.727	23.780	23.875	99820	359752	5.97	
38	23.875	23.907	23.960	14676	54025	0.90	
39	23.960	24.087	24.150	28888	120928	2.01	
40	24.214	24.267	24.319	12050	44994	0.75	

Chromatogram Peaks

Peak	StartRT	RT	EndRT	Height	Area	AreaPct	SNR
41	24.319	24.393	24.638	35015	162404	2.70	
42	24.691	24.774	24.827	12793	47240	0.78	
43	24.827	24.880	24.912	8123	27172	0.45	
44	24.912	24.996	25.028	9012	34541	0.57	
45	25.028	25.070	25.208	87382	229216	3.81	
46	25.208	25.282	25.373	23656	81580	1.35	
47	25.493	25.567	25.684	8195	46179	0.77	
48	25.684	25.779	25.858	7113	29840	0.50	
49	26.196	26.287	26.421	7484	35525	0.59	
50	27.178	27.228	27.281	101943	241878	4.02	
51	27.281	27.344	27.482	287438	700048	11.62	
52	27.587	27.640	27.693	32676	83495	1.39	
53	27.709	27.862	27.905	12125	51570	0.86	
54	27.905	27.947	28.007	69261	164628	2.73	
55	28.032	28.074	28.127	13554	32501	0.54	
56	28.127	28.180	28.307	50250	138289	2.30	
57	28.518	28.582	28.705	32096	107959	1.79	
58	28.993	29.036	29.100	97364	242634	4.03	
59	29.288	29.354	29.555	192845	803821	13.34	
60	29.639	29.671	29.798	6847	44720	0.74	
61	29.798	29.830	29.946	5424	34428	0.57	
62	29.946	29.999	30.113	15833	51233	0.85	
63	30.315	30.358	30.439	51364	134378	2.23	
64	31.046	31.131	31.183	11145	37245	0.62	
65	31.183	31.268	31.300	6114	23714	0.39	
66	31.430	31.511	31.585	2049222	5330616	88.49	
67	31.585	31.628	31.815	69917	265234	4.40	
68	32.008	32.051	32.257	30841	128571	2.13	
69	32.583	32.622	32.700	33689	96241	1.60	
70	32.780	32.886	33.468	87164	988432	16.41	
71	33.961	34.029	34.081	204696	557918	9.26	
72	34.081	34.124	34.198	112969	353991	5.88	
73	34.198	34.240	34.346	22379	74265	1.23	
74	34.492	34.547	34.615	29513	82355	1.37	
75	35.118	35.192	35.382	75165	382408	6.35	
76	35.382	35.414	35.696	9065	61494	1.02	
77	42.913	44.065	44.235	8062	750082	12.45	

Compound Summary

Cpd	CpdName	Formula	RT	Mass	CAS	IDSourceBest	Score_ID	Score_Lib	Score_DB	Score_MFG	Algorithm
1	.beta.-Pinene	C10 H16	8.190		127-91-3	Lib searching	95.22	95.22			integral
2	Methyl salicylate	C8 H8 O3	14.219		119-36-8	Lib searching	85.13	85.13			integral
3	?		17.328			Lib searching	100.00				integral
4	δ-Elemene	C15 H24	17.540		20307-84-0	Lib searching	100.00				integral
5	isoledene	C15 H24	18.344		95910-36-4	Lib searching	92.39	92.39			integral
6	β-Elemene	C15 H24	18.767		515-13-9	Lib searching	100.00				integral
7	1H-Cycloprop[e]azulene, 1a,2,3,4,4a,5,6,7b-octahydro-1,1,4,7-tetramethyl-, [1aR-(1a.alpha.,4.alpha.,4a.beta.,7b.alpha.)]-	C15 H24	19.158		489-40-7	Lib searching	95.08	95.08			integral
8	?		19.253			Lib searching	100.00				integral
9	Caryophyllene	C15 H24	19.370		87-44-5	Lib searching	99.32	99.32			integral
10	Calarene	C15 H24	19.549		17334-55-3	Lib searching	100.00				integral
11	α-Guaiene	C15 H24	19.687		3691-12-1	Lib searching	100.00				integral
12	Aromandendrene	C15 H24	19.782		489-39-4	Lib searching	98.46	98.46			integral
13	Selina-5,11-diene	C15 H24	19.867		52026-55-8	Lib searching	100.00				integral
14	Humulene	C15 H24	20.089		6753-98-6	Lib searching	94.79	94.79			integral
15	Alloaromadendrene	C15 H24	20.184		25246-27-9	Lib searching	100.00				integral
16	γ-Muurolene	C15 H24	20.522		30021-74-0	Lib searching	100.00				integral
17	γ-Selinene	C15 H24	20.575		515-17-3	Lib searching	100.00				integral
18	trans-.beta.-Ionone	C13 H20 O	20.766		79-77-6	Lib searching	79.84	79.84			integral
19	β-Guaiene	C15 H24	20.819		88-84-6	Lib searching	100.00				integral
20	(1S,2E,6E,10R)-3,7,11,11-Tetramethylbicyclo[8.1.0]undeca-2,6-diene	C15 H24	20.988		24703-35-3	Lib searching	98.06	98.06			integral
21	α-Farnesene	C15 H24	21.199		502-61-4	Lib searching	100.00				integral
22	γ-Cadinene	C15 H24	21.337		39029-41-9	Lib searching	100.00				integral
23	Nootkatene	C15 H22	21.400		5090-61-9	Lib searching	100.00				integral
24	Naphthalene, 1,2,3,5,6,8a-hexahydro-4,7-dimethyl-1-(1-methylethyl)-, (1S-cis)-	C15 H24	21.506		483-76-1	Lib searching	94.12	94.12			integral
25	(8R,8aS)-8,8a-Dimethyl-2-(propan-2-ylidene)-1,2,3,7,8,8a-hexahydronaphthalene	C15 H22	21.739		27840-40-0	Lib searching	85.09	85.09			integral
26	1,6,10-Dodecatrien-3-ol, 3,7,11-trimethyl-, (E)-	C15 H26 O	22.289		40716-66-3	Lib searching	95.32	95.32			integral
27	(1aR,3aS,7S,7aS,7bR)-1,1,3a,7-Tetramethyldecahydro-1H-cyclopropa[a]naphthalen-7-ol	C15 H26 O	22.416		527-90-2	Lib searching	83.55	83.55			integral
28	1H-Cycloprop[e]azulen-7-ol, decahydro-1,1,7-trimethyl-4-methylene-, [1ar-(1a.alpha.,4a.alpha.,7.beta.,7a.beta.,7b.alpha.)]-	C15 H24 O	22.627		6750-60-3	Lib searching	97.16	97.16			integral

Analysis Report

Compound Summary

Cpd	CpdName	Formula	RT	Mass	CAS	IDSourceBest	Score_ID	Score_Lib	Score_DB	Score_MFG	Algorithm
29	(-)-Globulol	C15 H26 O	22.754		489-41-8	Lib searching	91.69	91.69			integral
30	Guaiol	C15 H26 O	22.913		489-86-1	Lib searching	100.00				integral
31	Humulene epoxide I	C15 H24 O	23.018		19888-33-6	Lib searching	100.00				integral
32	2-Naphthalenemethanol, 2,3,4,4a,5,6,7,8-octahydro-.alpha.,.alpha.,4a,8-tetramethyl-, [2R-(2.alpha.,4a.beta.,8.beta.)]-	C15 H26 O	23.114		63891-61-2	Lib searching	84.10	84.10			integral
33	Humulene epoxide 2	C15 H24 O	23.241		19888-34-7	Lib searching	100.00				integral
34	?		23.484			Lib searching	100.00				integral
35	Ledene oxide-(II)	C15 H24 O	23.579		882187-44-2	Lib searching	100.00				integral
36	Caryophylladienol II	C15 H24 O	23.685		38284-26-3	Lib searching	100.00				integral
37	Isospathulenol	C15 H24 O	23.780		88395-46-4	Lib searching	100.00				integral
38	α-Vetivol	C15 H24 O	23.907		57422-86-3	Lib searching	100.00				integral
39	Neointermedeol	C15 H26 O	24.087		5945-72-2	Lib searching	100.00				integral
40	Ylangenol	C15 H24 O	24.267		41610-69-9	Lib searching	100.00				integral
41	Aromadendrene epoxide	C15 H24 O	24.393		94020-95-8	Lib searching	100.00				integral
42	Acorenone B	C15 H24 O	24.774		21653-33-8	Lib searching	100.00				integral
43	?		24.880			Lib searching	100.00				integral
44	?		24.996			Lib searching	100.00				integral
45	Pentadecanal-	C15 H30 O	25.070		2765-11-9	Lib searching	95.52	95.52			integral
46	trans-Farnesol	C15 H26 O	25.282		106-28-5	Lib searching	100.00				integral
47	Farnesol ,2E, 6Z-	C15 H26 O	25.567		3879-60-5	Lib searching	100.00				integral
48	?		25.779			Lib searching	100.00				integral
49	Anthracene	C14 H10	26.287		120-12-7	Lib searching	67.78	67.78			integral
50	Neophytadiene	C20 H38	27.228		504-96-1	Lib searching	95.24	95.24			integral
51	2-Pentadecanone, 6,10,14-trimethyl-	C18 H36 O	27.344		502-69-2	Lib searching	97.66	97.66			integral
52	3,7,11,15-Tetramethyl-2-hexadecen-1-ol	C20 H40 O	27.640		102608-53-7	Lib searching	91.27	91.27			integral
53	Benzoic acid, 2-hydroxy-, phenylmethyl ester	C14 H12 O3	27.862		118-58-1	Lib searching	64.74	64.74			integral
54	E-11-Hexadecen-1-ol	C16 H32 O	27.947		61301-56-2	Lib searching	100.00				integral
55	8,11-Heptadecadienal, (8Z,11Z)-	C17 H30 O	28.074		56797-42-3	Lib searching	100.00				integral
56	Methyl (7E,10E)-7,10-hexadecadienoate	C17 H30 O2	28.180		17364-31-7	Lib searching	100.00				integral
57	Farnesyl acetone	C18 H30 O	28.582		1117-52-8	Lib searching	100.00				integral
58	Isophytol	C20 H40 O	29.036		505-32-8	Lib searching	96.71	96.71			integral
59	n-Hexadecanoic acid	C16 H32 O2	29.354		57-10-3	Lib searching	100.00				integral
60	?		29.671			Lib searching	100.00				integral
61	?		29.830			Lib searching	100.00				integral
62	13-Octadecenal	C18 H34 O	29.999		56554-90-6	Lib searching	100.00				integral
63	1,6,10,14-Hexadecatetraen-3-ol, 3,7,11,15-tetramethyl-, (E,E)-	C20 H34 O	30.358		1113-21-9	Lib searching	92.87	92.87			integral
64	9-Octadecenenitrile, (Z)-	C18 H33 N	31.131		112-91-4	Lib searching	77.44	77.44			integral
65	?		31.268			Lib searching	100.00				integral
66	2(3H)-Furanone, 5-dodecyldihydro-	C16 H30 O2	31.511		730-46-1	Lib searching	94.75	94.75			integral
67	Phytol	C20 H40 O	31.628		150-86-7	Lib searching	89.95	89.95			integral
68	Oleic Acid	C18 H34 O2	32.051		112-80-1	Lib searching	100.00				integral
69	1-Docosene	C22 H44	32.622		1599-67-3	Lib searching	100.00				integral
70	Phenol, 4,4'-(1-methylethylidene)bis-	C15 H16 O2	32.886		80-05-7	Lib searching	93.14	93.14			integral
71	Methyl 5,11,14-eicosatrienoate	C21 H36 O2	34.029		97016-84-7	Lib searching	100.00				integral
72	(Z)-9-octadecen-4-olide	C18 H32 O2	34.124		59303-50-3	Lib searching	100.00				integral
73	11,14-Eicosadienoic acid, methyl ester	C21 H38 O2	34.240		2463-02-7	Lib searching	100.00				integral
74	2(3H)-Furanone, dihydro-5-tetradecyl-	C18 H34 O2	34.547		502-26-1	Lib searching	83.69	83.69			integral
75	Octadecanamide	C18 H37 N O	35.192		124-26-5	Lib searching	100.00				integral
76	Oleamide	C18 H35 N O	35.414		301-02-0	Lib searching	100.00				integral

MassHunter Qual 10.0
(End of Report)
